# Battle between Host Immune Cellular Responses and HCMV Immune Evasion

**DOI:** 10.3390/ijms20153626

**Published:** 2019-07-24

**Authors:** Trishna Manandhar, Gia-Gia T. Hò, Wiebke C. Pump, Rainer Blasczyk, Christina Bade-Doeding

**Affiliations:** Institute for Transfusion Medicine, Hannover Medical School, 30625 Hannover, Germany

**Keywords:** HCMV, immune evasion mechanisms, immune surveillance mechanisms, virus–host interaction

## Abstract

Human cytomegalovirus (HCMV) is ubiquitously prevalent. HCMV infection is typically asymptomatic and controlled by the immune system in healthy individuals, yet HCMV can be severely pathogenic for the fetus during pregnancy and in immunocompromised persons, such as transplant recipients or HIV infected patients. HCMV has co-evolved with the hosts, developed strategies to hide from immune effector cells and to successfully survive in the human organism. One strategy for evading or delaying the immune response is maintenance of the viral genome to establish the phase of latency. Furthermore, HCMV immune evasion involves the downregulation of human leukocyte antigens (HLA)-Ia molecules to hide infected cells from T-cell recognition. HCMV expresses several proteins that are described for downregulation of the HLA class I pathway via various mechanisms. Here, we review the wide range of immune evasion mechanisms of HCMV. Understanding the mechanisms of HCMV immune evasion will contribute to the development of new customized therapeutic strategies against the virus.

## 1. Human Cytomegalovirus (HCMV)

Human cytomegalovirus (HCMV), a human herpesvirus-5 (HHV-5), belongs to β-herpesviridae subfamily within herpesviridae. The characteristic appearance of HCMV infected cells with swollen, granular filled cell bodies were first described by Ribbert in 1881 [1]. Until 1921, it was suggested by Goodpasture and Talbert that the reason for the cellular changes is a virus [2]. Finally, human cytomegalovirus (HCMV) was isolated from cell culture by three independent researchers [3,4,5].

HCMV is ubiquitously prevalent and infects approximately 60% of adults in developed countries and 100% in developing countries [6,7]. A latent HCMV infection in immune competent individuals can be controlled by the immune system [8] with no pathology. HCMV infection is typically asymptomatic in healthy individuals, ~10% develop HCMV mononucleosis [9]; however HCMV can be severely pathogenic in a few groups: a fetus during pregnancy and immunocompromised persons, especially HIV infected patients and transplant recipients. In HIV-infected individuals, the degree of immunosuppression correlates with the risk of developing symptomatic HCMV disease. The occurrence of HCMV disease increases especially if the CD4^+^ T cell count drops below 100 cells/µL [10,11,12]. The congenital infection of the fetus is dependent on the vertical transmission of HCMV from the mother infected with the virus previously or during the current pregnancy [13]. The infants born with HCMV infection are at high risk of long-term health disabilities, particularly neurological and sensory impairments [14]. The overall risk of children born with HCMV is estimated at 0.7%. Approximately 12.7% of the infected children developed HCMV-specific syndromes at birth [15,16]. Beside the congenital infection, HCMV is associated with several circumstances of mortality and morbidity; including as a common opportunist in solid organ and bone marrow transplant recipients, a significant contributor to mortality in HIV infected patients and furthermore, HCMV might be a cause of immunosenescence in the elderly population [6]. The definition of HCMV infection and HCMV disease has been updated recently by the CMV Drug Development Forum [17]. An HCMV infection means that virus could be isolated or detected; a recurrent HCMV infection means that a new HCMV infection could be detected in a patient with previous evidence of HCMV infection, this recurrent infection could be a result of reactivation of latent virus (endogenous) or reinfection (exogenous); an HCMV disease means the diagnosis of HCMV infection in a patient with manifested symptoms.

## 2. Implications of HCMV in Hematopoietic Stem Cell Transplantation (HSCT)

HCMV is an important factor associated with mortality and transplant related complications following hematopoietic stem cell transplantation (HSCT). Although the available antiviral drugs (ganciclovir, forscarnet and cidofovir) reduced the mortality caused from early HCMV effects, the reduction of mortality from late HCMV diseases still remains questionable [18]. The direct clinical effects of HCMV infections on patients post HSCT include pneumonia, gastrointestinal diseases, retinitis and CNS disorders [19,20,21]; the indirect manifestation include accelerated arthrosclerosis and bacterial or fungal super-infections. A bidirectional link between HCMV and graft versus host disease (GvHD) has been observed. Studies show that GvHD and its treatment regimens can increase the risk of HCMV infections in the transplant recipients [22]. Simultaneously, some studies suggest that HCMV infections lead to the severity of GvHD in transplant recipients [23,24]. The serological status of HCMV in donor and recipient plays an important role in determining the clinical manifestation by HCMV following HSCT, the seropositive recipient posing the highest risk [8,25]. An HCMV seropositive donor is associated with increased mortality and other secondary infections in the recipient of a stem cell transplant compared to the HCMV seronegative donor [26,27].

## 3. Bidirectional Relationship between Host Strategies and HCMV Immune Escape Mechanisms

Continuous battle between human immune surveillance and HCMV immune evasion is observed in the process of natural selection. HCMV immune evasion involves the downregulation of HLA-Ia molecules to hide infected cells from T-cell recognition. HLA-Ia downregulation goes usually along with the up-regulation of HLA-Ib molecules to confer tolerance to HLA-Ia negative cells from recognition by natural killer (NK) cells. The involvement of HLA-Ia/Ib molecules for HCMV infected cells to become invisible for the immune system is an elaborate strategy and makes the combat of this infection very complicated. However, CD8^+^ T cells, the key players in adaptive cellular immunity, provide long-lasting protection. The viral peptides presented on HLA molecules are recognized by these effector cells, hence, augmenting proper immune response. Available peptides are selected and loaded onto HLA molecules with the assistance and chaperoning of certain highly specialized proteins, the peptide loading complex (PLC). This is an assembly of several macromolecular structures including calreticulin (CRT), thiol-dependent ERp57 (PDIA3), the two subunits of the transporter associated with antigen processing (TAP) and the HLA class I dedicated tapasin (TPN), a protein that assists in selecting and loading optimized peptide ligands in the HLA peptide binding groove. Peptides that origin from proteolytic degradation of intracellular proteins [28] are translocated from the cytosol into the endoplasmic reticulum (ER) lumen by the assistance of TAP [29,30]. The ER aminopeptidases 1 and 2 (ERAP1 and ERAP2) trim these peptides in the ER lumen further. HLA class I folding and assembling of nascent molecules occur in the ER with the chaperoning of calnexin (CNX), CRT and ERp57 [31,32]. By transient interaction with the proteins of the PLC, the HLA heavy chain (hc) and beta-2-microglobulin (β_2_m) attain a peptide receptive form; peptides are then loaded into the peptide binding region PBR with the assistance of TPN. Finally, peptide-HLA (pHLA) complexes disintegrate from the PLC and are translocated to the cell surface where they are available for the survey by immune effector cells [32,33].

Presentation of viral peptides on HLA molecules is a crucial event for recognition of viral infection and its resolution by immune effector cells. Downregulation of the antigen presentation pathway, therefore, has major impact on the loading of optimized peptides, hence, affecting the whole phenomenon of immune recognition. HCMV aims to inhibit the components of the PLC with the ultimate aim to prevent the presentation of viral peptides. However, in the course of evolution, some HLA alleles are evolved to fight back these immune evasions by choosing pathways [34,35,36] that could operate independently of the PLC components. There are certain key polymorphisms within the hc of HLA molecules that can actually alter the mode of peptide loading. For instance, a single amino acid (aa) mismatch at position 116 or 156 can confer TPN-independence.

## 4. HCMV Virus Genome and Gene Expression 

HCMV virion consists of an outer membrane envelope, a phosphoprotein tegument layer and a capsid enclosing viral genome. The outer membrane includes several glycoproteins that serve the function of viral attachment. The HCMV genome is an approximately ~240 kbp linear, double-stranded DNA molecule encoding more than 700 potential open reading frames (ORFs) [37]. The genome comprises of two major regions, the unique long (UL) and unique short (US) segments. Each UL and US segment is flanked by two sets of inverted DNA repeats that are denoted by the internal/terminal repeats long (IRL/TRL) or internal/terminal repeats short (IRS/TRS). The simplified structure of HCMV genome can be represented as TRL–UL–IRL–IRS–US–TRS [38].

**Figure 1:**
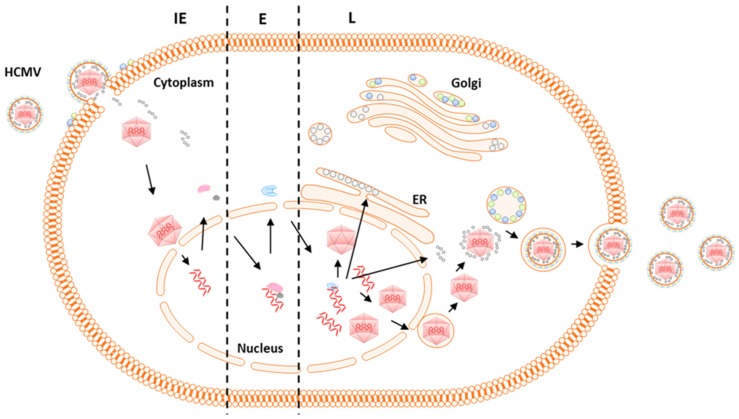
Human cytomegalovirus (HCMV) life cycle. HCMV enters the cell through interaction of the host receptors with specific viral glycoproteins. Capsid and tegument proteins are release into the host cytosol. The capsid releases the viral genome into the nucleus, leading to the expression of immediate early (IE) genes. The IE proteins activate the expression of the early (E) genes. The E proteins initiate viral genome replication and the expression of late (L) genes. The L gene expression initiates the capsid assembly and the expression of tegument- and glycoproteins. The genome-loaded capsid enters the cytosol via nuclear egress. The capsid associates with the tegument proteins. The capsid acquires the viral envelope by budding into intracellular vesicles. The enveloped viral particles are released into the extracellular space.

HCMV can infect a broad range of host cells including monocytes/macrophages, fibroblasts, epithelial, endothelial and dendritic cells [39]. The attachment of HCMV to the host cells occurs as a result of the interaction of host receptors with specific viral glycoprotein complexes, gB, gH/gL/gO or pentameric gH/gL/UL128/130/131 [40,41,42]. After the attachment of virus to the host cell the alpha helical coils, formed from heptad repeat regions in gB and gH, cause membrane fusion and release of viral capsids into the host cytosol [43,44]. Transcription of HCMV genes occurs in a temporally regulated cascade. The productive infection characterized by replicative cycles of three chronological phases: immediate early (IE), early (E), and late (L) [45,46,47]. The IE genes are first to be transcribed independently of any de novo host or viral protein synthesis and function as transcription factors of early proteins. Most of the early HCMV genes play an essential role in viral replication. Proteins from the late genes serve as structural components of virions and are necessary in virion assembly and egress (Figure 1).

**Figure 2:**
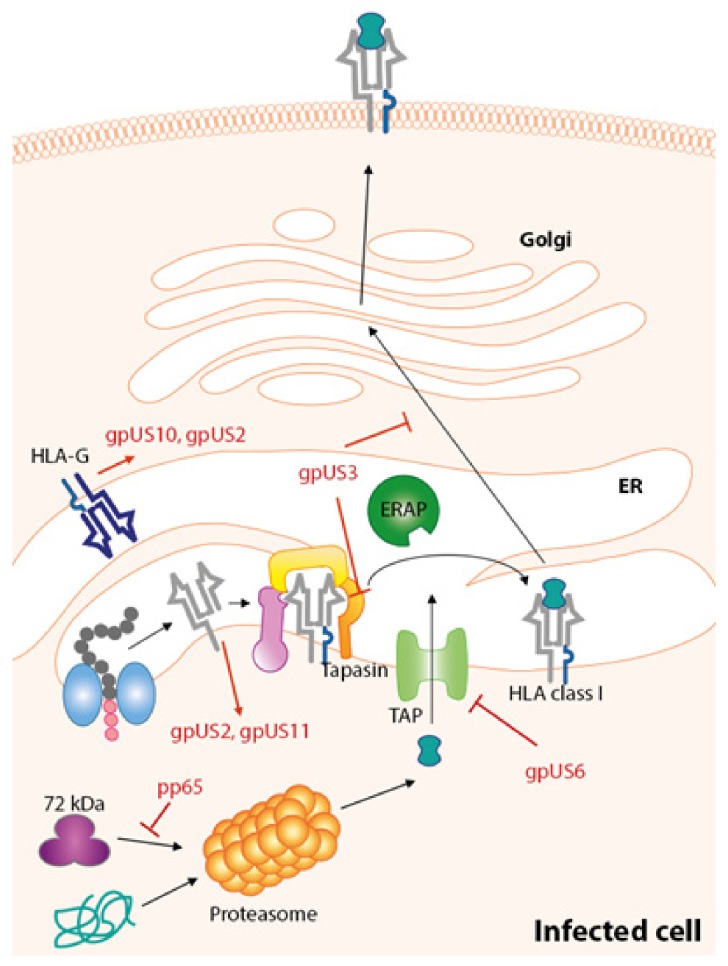
HCMV-mediated downregulation of HLA-class I pathway. The protein pp65 prevents the presentation of IE-derived peptides. Glycoprotein unique short 2 (gpUS2) and US11 (gpUS11) redirect HLA class I heavy chain (hc) from the endoplasmic reticulum (ER) to the cytosol and induce the proteasomal degradation of the molecule. Glycoprotein US6 (gpUS6) inhibits the function of transporter associated with antigen processing (TAP). Glycoprotein US3 (gpUS3) inhibits the tapasin-dependent peptide loading and retains HLA class I molecules in the endoplasmic reticulum (ER). Glycoprotein US10 (gpUS10) retains HLA class I hc in the ER. gpUS10 and gpUS2 induce degradation of HLA-G.

The major IE proteins include IE1-pp72 (exons 1–4) and IE2-pp86 (comprising exons 1–3, 5), both are spliced products of the same IE transcript from the major IE region (UL122-123) [48,49,50]. The IE proteins transactivate or regulate the promoters of E genes independently as well as synergistically [51,52,53]. The IE2-pp86, in addition, can negatively autoregulate its own promoter by binding to the cis-repression signal [54,55,56]. IE2-pp86 also augments the HCMV replication and inhibits the expression of virus-induced chemokine following viral infection [57]. Beside the classical IE proteins, US3, US2 and US11 are also produced in the IE phase [58,59]; and these proteins serve as potent immune evasions (Figure 2).

## 5. Host Responses to HCMV Infection

### 5.1. Innate Immunity

NK cells and type I interferons (IFN-α/β) are classical innate immune players to provide antiviral effect against murine CMV (MCMV) replication [60,61]. Studies on NK cell deficient murine models and subsequent experiments with adoptive transfer of NK cells demonstrated the function of NK cells in controlling MCMV infections [62,63]. The role of NK cells in controlling HCMV in the context of human hosts has been described in several studies. Biron et al. [64] described the relationship between NK cell activity and HCMV via a case study of an HCMV susceptible NK cell deficient individual. Recent studies have shown the role of NK cells to inhibit HCMV transmission in fibroblasts, endothelial and epithelial cells by induction of IFN-β in infected target cells [65] and production of IFN-γ by NK cells [66]. Recently, donor activating killer Ig-like receptor genes that regulate NK cell function have been shown to impact on HCMV reactivation following HSCT [67,68]. The importance of toll like receptors 2 (TLR2) in innate immunity as sensors of HCMV envelope glycoproteins B (gB) and glycoprotein H (gH) has been demonstrated through the interaction of HCMV gB and gH with TLR2 on the plasma membrane of target cells that results in activation of the NF-κB pathway and production of inflammatory cytokines [69,70].

### 5.2. Adaptive Immunity

Adaptive immunity plays a crucial role in controlling the primary HCMV infection as well as maintaining a host–pathogen balance in the virus latency phase. Both humoral and cell mediated mechanisms provide a long-lasting immunity in healthy individuals by preventing acute viremia, productive infection and imposing HCMV latency. The effect of an impaired immune response can be observed in immunocompromised patients that can result in several CMV pathologies and mortality [71]. Adoptive transfer of HCMV-specific T cells has been considered a proven strategy to prevent HCMV disease following bone marrow transplantation [72,73,74,75]. However, following HCMV infection, aggravated HCMV specific cell mediated immunity could progressively alter the composition of the T cell repertoire by inducing a decrease of naïve T cells and an increase of highly differentiated T cells. The change in the homeostatic balance of cellular immunity could be responsible for premature immune senescence and cytotoxic T lymphocytes (CTL) associated vascular pathologies [76,77].

NK cells classically belong to the innate immune system; however, a linkage between innate and adaptive NK cells has been described [78,79] where NK cells in mice and humans were demonstrated to feature a broader diversity and antigen specificity. It could be demonstrated that mouse and human NK cells are selected based on their avidity to certain antigens during MCMV or HCMV infection [80].

#### 5.2.1. Humoral Immunity

Humoral immune responses to various HCMV epitopes are elicited in human hosts. A major role has been demonstrated for antibodies targeting the genes involved in endothelial/epithelial cell tropism [81,82]. However, the effect of humoral immunity in controlling HCMV infection is poorly understood. HCMV neutralizing antibodies isolated from serum of individuals with prior HCMV infection have shown to effectively block virus replication in endothelial/epithelial cells that use *UL131A–128* locus for virus entry, but not in fibroblasts which are infected via a different route of entry [81].

Women, who acquired primary HCMV infection during pregnancy (indicated by presence of IgM antibodies against HCMV) and lacking neutralizing IgG antibodies, are at higher risk of vertical transmission of HCMV to the fetus, compared to seropositive mothers getting a recurrent-infection [12,83]. Moreover, HCMV seropositivity was found to influence the lymphoid cellular repertoire and correlated with the magnitude of HCMV-specific T cell immune responses within healthy individuals [84]. However, epidemiological studies carried out between seropositive and seronegative elderly individuals reveal that the HCMV seropositivity could lead to clonal expansion of HCMV-specific CTLs and predispose individuals to immune senescence [85,86].

#### 5.2.2. Cellular Immunity

The conventional alpha beta (αβ) CD8^+^ T cells are the most studied and prominent effector cells to fight HCMV infections, however, recent studies shed light on various other T cell subsets that could be involved, like, CD4^+^ T cells and gamma delta (γδ) T cells. Characterization of T cell responses are typically done by stimulation of peripheral blood mononuclear cells (PBMC)s with peptide pools spanning key HCMV proteins, particularly confined to two important proteins: the 65 kDa matrix phosphoprotein (pp65), encoded by *UL83*, and the 55 kDa immediate early protein (IE-1), encoded by *UL123* [87]. However, the understanding of the repertoire of viral peptides potentially presented on HLA molecules and their effect on the host immune system is unclear. It has now been obvious that the T cell response to HCMV is broadly specific, comprising of HCMV proteins from all three phases of lytic infection (immediate-early, early and late) and all types of structural and non-structural HCMV proteins [88,89]. A cytokine flow cytometry assay with overlapping 15-mer peptides demonstrated that broadly targeted HCMV specific CD4^+^ and CD8^+^ T cells dominate the memory compartments of seropositive subjects [89].

In contrast to HCMV proteins engaged in lytic phase, less is known about the viral proteins that are expressed in latent infection and recognized by the host. Studies identified several latent infection associated HCMV proteins including UL138, LUNA (latency-associated unidentified nuclear antigen) an antisense transcript to the *UL81–82* region, UL111A (vIL-10) and US28 [90,91]. Ex vivo studies for healthy HCMV carriers demonstrated UL138 and LUNA-specific T cell responses, which are mostly led by CD4^+^ T cells [92]. A dominant CD8^+^ T cell response was demonstrated from healthy seropositive individuals for latency-associated pUL138 derived 13-meric peptide in association with HLA-B*35:01 [93].

##### HCMV-Specific CD8^+^ T Cell Responses

Adoptive transfer of HCMV-specific CD8^+^ T cells has been used as an approach to mitigate post transplantation HCMV related complications. Riddel et al. [72] demonstrated the first proof of principle that the transfer of donor derived CD8^+^ T cells could restore the antiviral immunity in immunodeficient patients following stem cell transplantation. These studies verifying the notion of adoptive T cell immunity became the base for several therapeutic strategies to control HCMV infections in HSCT recipients [75,94].

Virus-specific CD8^+^ T cell responses are often dependent on the avidity profiles of T cell receptors (TCR) to peptide-HLA (pHLA) complexes [95].

Peptides of pp65 andIE1 contain certain cytotoxic epitopes recognized by the TCRs of CD8^+^ T cells. Apart from these two proteins, it is possible that the HCMV immune evasion proteins could also harbor immunodominant epitopes that are likely to be presented by a given HLA molecule on the surface of the virus-infected cell and elicit cellular immunity [89].

Apart from TCR–pHLA profiles, the ex vivo characterization of the epitope-specific αβ TCR repertoire via single cell approach provides the insights on the relationship between the diversity of αβ TCRs and HCMV specific CTL mediated effector function [96]. Findings demonstrate the high prevalence of HCMV-specific complementarity-determining regions (CDR)-3αβ public motif usage and showed that αβ TCR diversity correlates inversely with HCMV-specific antibody levels [96], thus, emphasizing the importance of the αβ TCR repertoire in controlling HCMV pathogenesis.

Identification and characterization of epitopes recognized by virus-specific CTL are helpful to understand the dynamics of host immune responses and development of therapeutic strategies to enhance immunity against viral infections. Alkali hydrolysis of recombinant proteins and chromium release assays were used in past to identify HLA-restricted CTL epitopes [97]. The development of cytokine flow cytometry (CFC) aids in identification of peptide-specific IFN-γ expression in CD8^+^ T cells. [98]. Currently, rapid and efficient strategies for determination of CTL epitopes are based on in silico bioinformatics prediction followed by various ex vivo functional T cell assays (tetramers and IFN-γ-based ELISpot assays) [88,99].

##### HCMV-Specific CD4^+^ T Cell Responses

HCMV-specific CD4^+^ T cells provide immune protection by recognizing HCMV epitopes that are presented by HLA-class II molecules on the surface of antigen presenting cells (APCs). The lack of the helper response of CD4^+^ T cells is found to be associated with HCMV related complications [100,101]. Adoptive transfer of HCMV CD4^+^ T cells demonstrated the antiviral roles of virus-specific CD4^+^ T cells against murine CMV in immunocompromised mice [102,103]. Recently a subset of HCMV specific effector CD4^+^ T cells has been described that directly responses to the viral infection [104]. Moreover, CD4^+^ helper T cell function is required for the persistence of cytotoxic activity of adoptively transferred CD8^+^ T cells in recipients of allogeneic bone marrow transplants [105]. The transfer of HCMV-specific CD4^+^ T cells has been found to promote HCMV-specific CD8^+^ T cell expansion in stem cell transplant recipients [94].

Frequencies of HCMV-specific CD4^+^ T cells in seropositive subjects was 4.0% in peripheral blood and 9.1% in the memory compartment [89]. The CFC assay revealed that 151 (70%) of the 213 HCMV ORFs assessed were immunogenic for CD4^+^ T cells. Most cellular response studies for CD4^+^ T cells are also performed with pp65 andIE1 [106,107]. Nevertheless, other HCMV epitopes including those from many other ORFs including UL55 (gB), UL86, UL99, UL122 (IE-2), UL36, UL48 and IRS-1, are studied for their potential to elicit CD4^+^ T cell responses [89].

##### HCMV-Induced Regulatory T Cells (iTreg)

Regulatory T (Treg) cells are an important subpopulation of T cells that mediate immune suppression and homeostasis. The most important subset of Treg cells exhibit the biomarkers CD25^+^ CD4^+^ and express the transcription repressor forkhead box P3 (FOXP3). FOXP3^+^ Treg cells are involved in the suppression of development and activation of effector T cells, in conjunction with IL-2 [108]. There are two major types of regulatory T cells: natural Treg (nTreg) cells that develop in the thymus and induced Treg (iTreg) cells that are induced in the periphery by the antigen activation of naïve T cells [109]. HCMV specific iTreg cell responses were demonstrated by using peptide pools spanning the target proteins UL83, UL55, UL86, UL99, UL153 and UL32 [110]. Depletion of Treg cells from PBMCs leads to increased effector CD8^+^ T cell responses to HCMV pp65 antigen [111]. However, the exact role of Treg cells in immune regulation and homeostasis during the battle of HCMV and host defense still needs further investigation.

## 6. HCMV Escape Mechanisms

Co-evolution strategies provided the HCMV viruses persistence inside the host body and harbor from immune effector cells. One strategy for evading or delaying the immune response is maintenance of the viral genome to establish the phase of latency [112]. With this approach viruses can persist without the production of detectable infectious particles. However, the viruses can revert back to infectious condition under certain immunocompromised situations. The mechanisms of latency and reactivation in the case of HCMV is determined by the expression of IE genes and regulated by their transcriptional promoters collectively known as major IE promoters/enhancers [113]. Furthermore, HCMV genome encodes for different NK modulators, for example UL135, UL141, UL142 and UL148A, to inhibit NK cell activation and recognition [114].

Moreover, HCMV also uses the unique strategy to undermine the host anti-viral immunity through the process known as molecular mimicry. HCMV encodes for the proteins that are homologs of host immune-modulatory cytokines and their G protein-coupled receptors [115,116,117].

Another mechanism is HLA and peptide dependent mimicry, whereby the viral epitope mimics the host peptide. The peptide mimicry ensures the presentation of peptides without activation of immune effector cells [118,119].

Multiple pathways are targeted to evade the host immune system, one is the downregulation of HLA class I antigen presentation (Figure 2).

## 7. Immune Evasion Proteins Targeting HLA-Class I Pathway

Most of the known immune evasion proteins are encoded by the genes located in the US region of the HCMV genome. At least five ER resident glycoproteins (gpUS3, gpUS2, gpUS11, gpUS6 and gpUS10) are described for downregulation of HLA class I pathway via various mechanisms. There are further genes located in the HCMV genome that are responsible for class I downregulation. It was demonstrated that pp71, an immune evasion polypeptide encoded by *UL82* gene, interacts with the repressor of the major IE gene locus leading to an enhance expression of IE proteins, IE1-pp72 and IE-pp86 [120,121]. Although it was shown that the synthesized pp71 is able to suppress HLA class I pathway in glioblastoma cells [122], the actual role of pp71 in downregulation of immune system is poorly understood. Contrary to previous results, recent studies demonstrated the role of pp71 in enhancing the presentation of IE1-derived peptides by HLA class I molecules, thus supporting CD8^+^ recognition rather than CD8^+^ suppression [123]. The pp65 protein, encodes by *UL83,* inhibits antiviral gene expression and IFN signaling [124,125].

The bidirectional relationship between HCMV, immune evasions and host responses affects HCMV pathogenesis following solid organ and bone marrow transplantation. Expression levels of HCMV immune evasion genes, *US3, US6* and *US11*, were found to be independently correlated with HCMV associated post-transplant outcomes in solid organ transplant patients [126,127]. However, future research is required to address the understanding of the mechanisms underlying the effect of these immune evasion proteins on post transplantation outcomes, especially following HSCT.

### 7.1. gpUS3

gpUS3 immune evasion protein is produced by the HCMV infected cells in the IE stage of infection [58]. Jones et al. described, for the first time, that a gene locus between *US2* and *US11* contains genes responsible for downregulation of HLA class I pathway in a very early stage of infection [128]. gpUS3, alters the maturation of HLA class I heavy chains and retains HLA class I molecules in the ER [59,129,130]. Due to the only transient association of gpUS3 with HLA class I molecules and its shorter half-life, retained HLA class I molecules can still egress to the surface [130]. Not all HLA molecules could be equally affected by gpUS3 control. gpUS3 was shown to bind TPN and inhibit the interaction of TPN with the components of PLC, thus inhibiting the TPN dependent peptide loading on HLA class I molecules [131]. That leads to the suggestion that only TPN dependent HLA alleles could be affected by US3 and TPN independent alleles can selectively escape this immune evasion strategy. Moreover, gpUS3 could also alter the process of optimal peptide loading by modulating the protein disulfide isomerase (PDI), a chaperone that stabilizes a peptide-receptive site on HLA class I molecules [132]. Furthermore, gpUS3 works in co-operation with other immune evasion proteins to downregulate the HLA class I pathway. Studies demonstrated the collaboration of gpUS3 with gpUS11 or gpUS2 result in downregulation of HLA class I hc expression and enhanced retention of HLA class I complexes in the ER [133].

The in vitro experiments showed that the primary gpUS3 transcript undergoes the alternative splicing to generate three RNAs encoding 3.5, 17 and 22 kDa isoforms [134,135]. Full length gpUS3 protein is a 22 kDa protein and corresponds to 186 aa transcript. It consists of an ER luminal domain comprising N-linked glycan at Asn60, a transmembrane domain and a short cytoplasmic tail. The truncated 17 kDa isoform lacks the transmembrane domain. It was demonstrated that ER luminal domain of US3 is required for ER retention of itself while US3 mediated retention class I molecules in ER require both transmembrane domain and ER luminal domain of the 22 kDa [136]. Further investigations identified three ER luminal domain residues (Ser58, Glu63 and Lys64) as the key determinants for ER retention [137]. Immunoprecipitation experiments showed that both the luminal and transmembrane domains of gpUS3 are essential for its binding to class I molecules [136]. However, conflicting results were seen with NMR based predicted tertiary structure, where luminal domains of US3 do not physically interact with class I molecules in vitro [138]. The capability of gpUS3 protein to oligomerize through their ER luminal domains was described both in vitro in the predicted model and also in vivo, thereby suggesting that this phenomenon could account for its ER retention property [138]. Among the three gpUS3 proteins, only the full length 22 kDa gpUS3, but not the truncated 17 kDa gpUS3, has the ability to retain HLA molecules in the ER and physically associate with HLA hc [139,140]. The transmembrane segment, present only in a full length gpUS3, could also be critical for the immune evasion potential of gpUS3 protein. However, studies showed that the 17 kDa isoform competes with full-length US3 for binding to TPN and acts as a negative regulator of full-length gpUS3 activity [141].

### 7.2. gpUS2

gpUS2 was identified to destabilize the HLA class I molecules [142] and redirect the HLA class I hc from the ER to cytosol, where they are rapidly degraded by proteosomal enzymes [143]. Incorrectly folded proteins in the ER are recognized, dislocated and retrotranslocated to cytosol where they are finally degraded by the proteosomes. This process is called ER associated degradation (ERAD) and is essential to the HLA class I pathway. HCMV immune evasion proteins gpUS2 and gpUS11 take control over this process to degrade HLA class I molecules.

gpUS2 protein is expressed in the early to the late stage of HCMV infection. The major protein is a 22 kDa protein (199 aa residues) endoglycosidase H-resistant type-I membrane glycoprotein. Full-length gpUS2 contains an N-terminal 20 aa signal peptide followed by an ER luminal domain that includes a glycosylation site (Asn68). The ER luminal domain is followed by a disulfide bridge between Cys52 and Cys133, a 23 aa transmembrane domain (residues 162–185) and a short cytosolic tail (residues 186 to 199) [144].

The crystal structure of gpUS2 bound to the complex of the HLA-A*02:01, β_2_m and peptide was solved by Gewurz et al in 2001 (PDB:1IM3) [145]. This structure reveals that the luminal domain of gpUS2 folds in immunoglobulin like conformation and contacts the residues in the C-terminus of the HLA-A*02:01 molecule in the vicinity between α2 and α3. This interaction is located at the region which is not utilized by other components of PLC, thereby indicating the added advantage for gpUS2 to avoid the binding competition and invade the HLA molecules, already associating with PLC [146]. Moreover, the residues contacted by gpUS2 on HLA hc is only present on limited HLA molecules including certain species of HLA-A, HLA-B and HLA-G, that explains the HLA locus specific immune evasive activity of gpUS2 [147,148].

It was shown that gpUS2 uses the Sec61, an ER membrane translocator, to transfer the HLA class I molecules from the ER to cytosol [143]. The gpUS2 recruits the translocation of TRC8 enzyme, an ERAD E3 ligase, for ubiquitination of HLA-class I hc and their proteasomal degradation [149,150,151].

The crystal structure of gpUS2 could not resolve the gpUS2 N-terminal residues that form the signal peptide domain of gpUS2. Unlike US11, whose signal peptide is cleavable [152], gpUS2 contains a non-cleavable signal peptide at its N-terminus [153]. However, it was found that the signal peptide peptidase (SPP) is required for gpUS2 mediated dislocation of HLA class I molecules [154]. The role of SPP in the ERAD pathway is to cleave the signal peptide from proteins in Sec61 translocon associated dislocation [155]. However, the exact role of SPP, its association with TRC8 and SPP/TRC8 hub is poorly understood in gpUS2 mediated degradation. Moreover, PDI, an ER resident chaperone, was found to be essential for SPP mediated degradation of proteins by US2 [156].

The transmembrane and cytosolic tail of the gpUS2 is essential for HLA dislocation and degradation. The truncated mutant of gpUS2, lacking the cytosolic tail, was unable to induce the degradation of HLA hc [144,157]. The US2 cytoplasmic tail is essential for TRC8 ligase binding [149,158]. Studies demonstrated the crucial role of ATPase associated with various cellular activities (AAA) p97 ATPase in the US2 mediated translocation process [159]. The AAA p97 ATPase along with the dimeric adaptor complex, Ufd1-Npl4, forms a p97 ATPase dislocation complex, which recognizes the ubiquitin signal in proteins during translocation process [160]. The p97 ATPase works in assistance with several other delivery factors in targeting the proteins to proteasomal degradation [161,162].

### 7.3. US11

gpUS11 was shown to rapidly dislocate HLA class I hc from the ER to cytosol, where they are ultimately subjected to proteasomal degradation [163,164]. The immune evasion proteins gpUS11 and gpUS2 have overall similar functions to invade ERAD pathway. However, both of them can work independently of each other, differ significantly in structure and utilize different modes of action.

*US11* encodes a 215 aa long 32 kDa endoglycosidase H-sensitive, N-linked type-I membrane glycoprotein [163]. It is produced at the early stage of HCMV infection and persists throughout infection [165]. The full length of US11 consists of a 17 aa long signal peptide, a luminal domain (residues 18–182), a transmembrane domain (residues 183–203) and a cytoplasmic tail (residues 204–215). Like gpUS2, gpUS11 is able to interact with both free and HLA molecules associated to β_2_m [166]. Functional assays showed that the luminal domains of gpUS11 interact with the HLA hc at the peptide binding region [167,168].

The role of properly cleaved signal peptide is to ensure accurate positioning and processing of the proteins on the ER. Delayed cleavage of the gpUS11 signal peptide is observed and the rate of signal sequence cleavage depends on the residues located on the transmembrane domain [152,169]. Mutagenesis data show that the transmembrane domain of gpUS11 is critical for HLA molecules dislocation and egress [157,169]. Two residues on the transmembrane domain, Gln192 and Gly196, were found to be crucial for the US11 mediated degradation [168]. Disruptions of these residues result in decreased HLA dislocation [169].

In contrast to gpUS2, which depend on SPP/TRC8 degradation hub, HLA-class I hc degradation by gpUS11 is mediated by a Derlin-1 and transmembrane protein 129 (TMEM129) enzyme [170]. gpUS11 hijacks the TMEM129 enzyme for proteasomal degradation of HLA molecules. TMEM129 is a non-classical ERAD RING E3 Ligase that ubiquitinates HLA molecules in gpUS11 mediated degradation. gpUS11 cytosolic tail helps to prevent the TMEM129 mediated ubiquitination of gpUS11 itself [170]. A rhomboid pseudo-protease Derlin-1 acts as a bridge to link TMEM129 and transmembrane domain of gpUS11 via Gln192 [170,171]. In addition to transmembrane domain of US11 [171,172], cytosolic tail of the HLA hc was also found to play essential role in recruitment of Derlin-1 to HLA complex [173]. Moreover, it was demonstrated that gpUS11 also exploits other ubiquitin conjugating enzymes, including UBE2K (E2-25K) and UBE2J2, as its immune evasive strategy [174,175].

Following the ubiquitination of the HLA molecules, they are dislocated and subjected to proteasomal degradation. The dislocation complex of the gpUS11 mediated degradation was shown to consist of valosin-containing protein (VCP)-interacting membrane protein (VIMP) [172], the AAA ATPase p97 [159] and SEL1L [176].

The process of ubiquitination is poorly understood in case of gpUS11 mediated HLA hc dislocation and degradation. Several studies demonstrate the requirement of functional ubiquitin system for proteasomal degradation of HLA molecules by gpUS11 [177,178,179]. However, unlike gpUS2, ubiquitination of lysines or the N terminus of the class I heavy chain was not required in gpUS11 mediated dislocation [180]. Therefore, gpUS11 requires several ubiquitin related enzymes including E1, ubiquitin-activating enzyme. The role of these various ubiquitin related enzymes in gpUS11 mediated degradation is still ambiguous.

### 7.4. US6

gpUS6 was first described by Hengel et al. and Lehner et al. in 1997, for its ability to inhibit the function of TAP [181,182]. gpUS6 is produced in the late phase of HCMV infection [165]. It is a 21 kDa (183 aa) ER resident glycoprotein glycosylated at Asn52. It comprises of a signal peptide (residues 1–19), ER luminal domain (residues 20–144), transmembrane domain (residues 145–165) and a cytoplasmic tail (residues 166–183) [134].

There are several viral proteins that interfere with the function of TAP, including HCMV gpUS6, Herpes-simplex-virus (HSV)-1 ICP47, Epstein-barr-virus (EBV) BNLF2a and celloviral UL49.5. The nucleotide binding domains (NBD) of TAP1 and TAP2 consist of ATP binding motifs and are involved in the generation of energy for peptide translocation via ATP hydrolysis [183]. In the uninfected condition, peptide binding to TAP complex induce the phenomenon of dimerization and disintegration of the TAP NBD coupled with the process of ATP hydrolysis in these domains. This would generate the conformational changes in their transmembrane domains that finally facilitate the transport of cytosolic peptides across the membrane [183,184]. HCMV takes control over this normal phenomenon by recruiting gpUS6 to inhibit the conformational rearrangement of TAP following peptide binding [185]. Unlike HSV-1 ICP47 [186], HCMV US6 does not prevent peptide binding to TAP complex. Instead, it interferes with the ATP binding to NBD of TAP1, but not TAP2 [185,187]. ER luminal domains of US6 bind to at least four sites on heterodimeric TAP1/TAP2 complex [188,189]. The gpUS6 binding site on TAP1 and TAP2 is localized on terminal loops of the transmembrane domain (TMD) [189]. Multimeric complexes of US6 help to reach the distant binding targets at TMD domains of TAP1 and TAP2, thus interfering the crosstalk between TAP1 and TAP2 subunits [189].

Circular dichroism spectroscopy (CD) spectroscopy revealed the secondary structure of the active gpUS6 ER luminal domain comprising of 19% α-helixes, 25%, β-sheets and 27% β-turns [187]. Residues 89 to 108 of the HCMV US6 luminal domain interact with TAP, following the inhibition of the TAP, whereas other residues flanking this segment stabilize the interaction of TAP and gpUS6 [190].

### 7.5. US10

gpUS10 was identified for its ability to retain HLA class I molecules in the ER and delay the maturation of class I molecules [191]. gpUS10 is an ER resident membrane glycoprotein that associates with HLA class I hc [191]. Although gpUS10 showed physical interaction with HLA class I molecules, the interaction did not result in sufficient ER retention or downregulation of HLA molecules as observed for US11 and other known HCMV immune evasion proteins.

gpUS10 was described for its role in alteration of non-classical HLA class I molecules [192]. The immunoprecipitation experiments verified the physical association of gpUS10 with HLA-G. However, gpUS10 is involved in the downregulation of surface expression of HLA-G and thus interferes in HLA-G–mediated NK cell inhibition. Thus, degradation of HLA-G would result in NK cell activation. This mechanism could have special significance in support of HCMV infection during mother to fetus transmission of the virus.

## 8. Immune Evasions in Regulation of Effector Cell Activation

Apart from gpUS10 that degrade HLA-G, gpUS2 immune evasion was also demonstrated to interfere with the normal functioning of iron homeostasis related to the non-classical HLA class I molecule, human hemochromatosis protein (HFE) [193,194]. Two other genes within the US region in the HCMV genome, *US18* and *US20*, were recently described for their roles in interference with NK cell function. Gene deletion experiments demonstrated the potential role of these immune evasion proteins for lysosomal degradation of major histocompatibility complex class I related chain A (MICA), a known ligand for the NK cell activating receptor (NKG2D) [195].

Besides the genes in the US region of the HCMV genome, certain genes at the UL region were identified as being able to evade the host immune system by suppressing NK cell recognition [196]. While UL16, miR-UL112, UL83 act on NK cell activating receptors, UL40 and UL18 support the up-regulation of NK cell inhibitory receptors.

UL16 and miR-UL112, are described to suppress the NK cell activating receptor ligands (NKG2DL), thereby inhibiting the NK cell activity. UL16 prevents the surface expression of the NKG2DL including UL16 binding proteins 1 and 2 (ULBP1 and ULBP2) and MICB [197,198], whereas miR-UL112 downregulates the expression of MICB [199]. The tegument protein pp65, encoded by *UL83* gene, directly interacts with NK cell activating receptor NKp30 and aids the dislocation of CD3-zeta from NKp30, thus inactivating the NK signaling [200].

**Figure 3:**
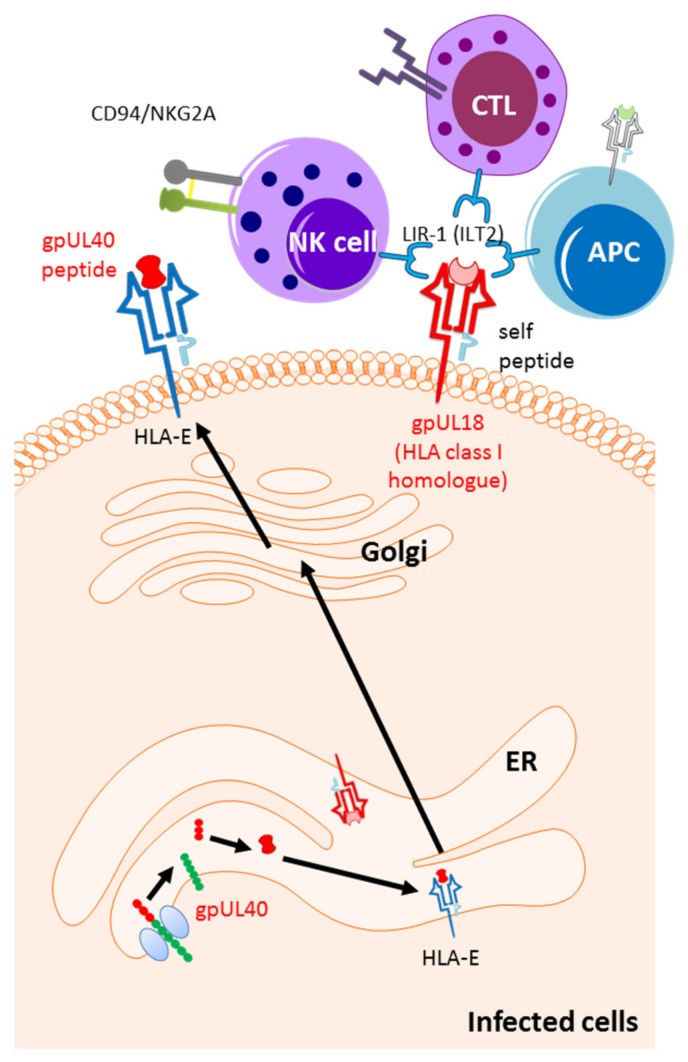
HCMV-mediated up-regulations of the ligands for natural killer (NK) cell inhibitory receptors. The signal peptide of unique long (UL)40 (gpUL40) up-regulate the surface expression of HLA-E. HLA-E is a ligand for the NK cell inhibitory receptor CD94/NKG2A. The HLA class I homologue, gpUL18, inhibits the NK cell activity by inducing NK cell inhibitory receptor LIR1 (ILT2).

UL40 contains the leader peptide specific for HLA-E. Presentation of UL-40 derived peptide on HLA-E would up-regulate NK cell inhibitory receptors CD94/NKG2A and prevents NK mediated lysis [201,202]. The surface expression of TAP dependent HLA–E specific peptides is required for activation of HLA-E [203]. However, when this process is arrested by the action of US6 that targets TAP, UL40 comes into action by augmenting HLA-E activity via the TAP independent presentation of UL-40 peptide on HLA-E [201,204]. Another protein, UL18, an HLA class I homologue, inhibits the NK cell activity by inducing NK cell inhibitory receptors LIR1 (ILT2) [205,206]. Moreover, UL18 acts together with gpUS6 to impede the interaction of HLA class I molecule with TAP [207] (Figure 3).

The laboratory adapted strain of HCMV with the deletion of UL/*b*′ sequence (genes *UL133*–*UL150*), AD169 and Towne, were found to be vulnerable to NK cell lyses compared to the low passage (Toledo) and wild strains [208,209]. These findings suggest that the genes located within HCMV UL/*b*′ region have NK cell modulatory functions. In this context, UL141 and UL142 has been shown to downregulate the expression of NK cell activating receptor ligands CD155 [209] and MICA [210] respectively. UL148A also targets MICA for lysosomal degradation [211]. MICA downregulation prevents the killing of HCMV-infected cells by NK cells. UL135 was reported to have a potential role in evading the NK cell mediated recognition via actin cytoskeleton remodeling [212]. UL148 suppresses the expression of co-stimulatory molecule CD58. The suppression of co-stimulation leads to the inhibition of CD8^+^ T cell and NK cell mediated antibody dependent cellular cytotoxicity [213].

## 9. Recent Advances in HCMV Therapeutics and Perspectives

Despite advancements made in the field of HSCT and viral infections, HCMV in particular, still remains a major problem that aggravates the post transplantation stages following immunosuppression regimes. HCMV infection, reactivation and HCMV disease in immunosuppressed patients can result in life-threatening situations [214]. The HCMV infection usually intensifies when T cell mediated cellular immunity is disrupted during immunosuppressive therapies. However, humoral immunity may play a role in disease severity. With the scientific progress in genetics, biotechnology and present knowledge of the HCMV genome, various HCMV targets show the potential to serve as the effective and safe therapeutic options to resolve HCMV related complications. Available pharmacological treatments of HCMV infections in immunocompromised patients include ganciclovir, valganciclovir, cidofovir, foscarnet and letermovir [215,216,217,218]. Despite improved outcomes, the emergence of resistance with prolonged use and the associated toxicities, restrict the pharmacological efficacy of these antiviral drugs. Several therapeutic strategies are being developed over the time to address the limitations associated with available antiviral agents and provide customized and optimal immune responses against HCMV immune evasions.

Adoptive transfer of donor-derived HCMV specific CTL is an attractive approach to rapidly restore virus-specific immunity to control HCMV infections after HSCT [105,219]. Efficacy of virus specific T cells in controlling viral infections depends on some fundamental subjects: effective mapping of potent T cell epitopes, expansion of virus-specific T cells and evaluation of the effector function of these T cells [220]. Published clinical trials employ HCMV specific CD4^+^ and CD8^+^ T epitopes belonging to the pp65 and IE1 proteins. HCMV-specific T cells are usually donor derived (generated from donor peripheral blood and donor stem cell harvest products) and could be expanded either via ex vivo expansion or direct selection with multimers or IFN-γ capture [221].

The HCMV lysate has been used as an activator for ex vivo expansion of HCMV specific T cells [94]. However, the use of viral lysates poses a potential infection risk of reactivation of the infectious virus. Some other studies use whole viral protein (HCMV-pp65) [74], recombinant pp65 protein [222] or dendritic cells pulsed with HCMV antigen [219] to produce CMV-specific T cells. In addition, HLA-restricted peptides, such as the HLA-A*02-restricted HCMV-pp65 epitope have been used as a target to stimulate T cell immunity [223,224].

However, in the face of HLA polymorphisms, use of a single epitope restricted to a specific HLA type, is not a sufficient therapeutic option. Recently, overlapping peptide pools or peptide mixtures derived from full-length HCMV target protein(s) have been evaluated in several preclinical and clinical studies [221,225,226,227,228].

Various vaccine strategies could serve a potential strategy to induce protective humoral immune response to CMV and the cellular immune response against HCMV. HCMV vaccine candidates, comprising live-attenuated HCMV vaccines, subunit vaccines, DNA vaccines, peptide vaccines and vector vaccines are currently undergoing investigation and a number of them are being evaluated in clinical trials [229,230]. Key targets of the HCMV specific T cell and B-cell immunity include envelope glycoproteins, particularly gB, the gH/gL-pentameric complex proteins, the pp65 and IE1 proteins. Glycoprotein B is recognized as a potent subunit protein vaccine, often used with an aluminum hydroxide or MF59 adjuvant, to enhance the immune responses against HCMV [231,232]. The HCMV DNA vaccines expressing gB, pp65 and IE1 in monovalent, bivalent or trivalent formulations are being investigated for clinical trials [229]. Several peptide immunogens comprising of short peptide fragments of HCMV origin have been engineered and evaluated for their potential use as peptide vaccines and to provoke the highly targeted immune responses [233,234]. Epitope mapping, enabling the rapid identification of cytotoxic epitopes, have been mainly focused on the pp65, IE1 and IE2 antigens [233]. Beside this, a number of antigenic domains within gB (AD-1, AD-2, AD-5) are potent B cell epitopes and shown to provoke the HCMV specific neutralizing antibodies. The AD-2 located on the N-terminus of gB has been evaluated in pre-clinical studies [235].

## 10. Conclusions

Structural analysis of immune evasion proteins would be the key to understand the interaction of the immune evasion proteins with other several accessory host/viral proteins and the molecular mechanisms of HCMV pathogenesis. Future studies are required to understand the underlying basis of the co-evolutionary battle between HLA polymorphism and emerging immune evasion proteins. For instance, investigation of impact of HCMV immune evasions on the repertoire of presented antigenic peptides on polymorphic HLA molecules would shed light on role of HLA polymorphisms and their implication on immune evasions in the context of HSCT. The outcomes of these studies could provide further clues to support novel customized therapeutic strategies against HCMV immune evasion strategies.
